# Tourism at the Crossroads between Well-Being, Public Health and the Environment: Panel Data Evidence from the European Union

**DOI:** 10.3390/ijerph191912066

**Published:** 2022-09-23

**Authors:** Daniel Badulescu, Ramona Simut, Ciprian Simut, Andrei-Vlad Badulescu

**Affiliations:** 1 Department of Economics and Business, Faculty of Economic Sciences, University of Oradea, 410087 Oradea, Romania; 2 Department of Philosophy, Nelson Mandela University, Port Elizabeth 6031, South Africa; 3 Faculty of Psychology and Educational Sciences, Education, Reflection, Development Doctoral School, Babes-Bolyai University, 400000 Cluj-Napoca, Romania; 4 Faculty of Medicine, Iuliu Hatieganu University of Medicine and Pharmacy, 400012 Cluj-Napoca, Romania

**Keywords:** health, well-being, environment, happiness, international tourism

## Abstract

The recent pandemic crisis led to a drop in tourism, and it highlighted the connection between tourism, healthcare, environmental concerns and well-being. In this context, the purpose of the research is to clarify the relationship between tourism, happiness, healthcare and environmental expenditure. Statistical data provided by the World Bank, Eurostat and the World Happiness Database from the EU27 countries, from 2000 to 2019, were used. In order to investigate the relationship between these indicators, the panel Autoregressive Distributed Lag (ARDL) method was used. In the long run, happiness and environmental and healthcare expenditure have a statistically significant and positive impact on tourism arrivals and receipts. It follows that a 1% increase in happiness supports between 4% and 9% of international tourism, while a 1% increase in environmental expenditure supports an increase of 2% in international tourism. Additionally, there is a significant interaction between happiness and either environmental or healthcare expenditure in the long run. This means that increasing happiness diminishes the effect of the later on tourism arrivals and receipts. No short-term relationship was identified between arrivals and any of the above-mentioned variables. In the same context, healthcare expenditure has a negative short-term effect on tourism receipts. The research contributes to the literature by suggesting that increasing national happiness, healthcare and environmental expenditure has a beneficial spillover effect on tourism arrivals and receipts in the long run.

## 1. Introduction

Tourism, considered the largest industry in the world and providing cca. 10.3% of all jobs (333 million persons) and 10.3% of global GDP (USD 9.6 trillion) [1,2], is now facing considerable opportunities, as well as challenges. While predictions made more than 3 years ago, in 2019, suggested that international tourism arrivals would increase in 2020 to 1.6 billion, and that “1.8 billion is now likely to be exceeded in advance of 2030” [3], the global pandemic crisis, followed by the energy and the political shocks of 2021–2022, significantly tempered these optimistic scenarios. Tourist flows dropped dramatically in 2020 (by cca. 75% globally) and slightly rebounded in 2021, but, despite all the challenges, experienced rapid growth in mid-2022 [4]. It is likely that catching up to and surpassing the records of 2017–2019 will occur in the mid-2020s, but challenges and risks remain, and they seem to diversify. It could be stated that the tourism industry, healthcare and environmental protection officials only now begin to understand the challenges entailed by such a rapid growth.

Is tourism growth sustainable and favorable to the environment, public health and well-being? Is there a relationship between environmental expenditure and public health, happiness and tourism flows? If so, can governments improve them and use them for the benefit of the citizens, to contribute to (socially and economically) more profitable—and more sustainable—tourism? These are some of the main questions that motivated the present paper.

The main purpose of the research is to clarify the relationship between tourism, happiness, healthcare, and environmental expenditure. Our objectives were to identify the long- and short-term relationship between international tourism and the three previously mentioned factors, at the level of the 27 European Union (EU) countries, by using panel data analysis. We attempted to fill an existing gap in the literature by taking into account these predictors in a multivariate model, as well as trying to understand the interactions between the predictors, namely, whether the effects of environmental and healthcare expenditure on tourism are mediated by happiness.

We hope that our study will prove helpful to researchers and practitioners in tourism, public health and environmental protection, but also to decision makers in defining policies that would exploit the synergies between happiness, healthcare and environmental expenditure as assets to increase tourism revenues. The novelty of the research is constituted by the attempt to identify the connections and the consequences between tourism, on the one hand, and happiness, healthcare and environmental expenditure, on the other, but with input from national governments. The involvement of the government presents itself as a challenge for the tourism industry, which this paper engages in analyzing. The contribution of the research is related to the identification of several long- and short-term consequences of various policies on tourism, especially in relation to factors such as happiness, and health and environmental expenditure. The problem presented by the research revolves around the measures by which tourism can be transformed into a more humane, culturally friendly and supportive industry that would engage in a sustainable manner with local cultures—bridging the cultural gap between world cultures—and promote itself as a health factor and facilitator; this would create a system that supports health, and collaborates at national levels with governments’ expenditure in matters of health and the environment. The answers to the research questions, starting from the issues mentioned above, converge on showing the importance of governmental health and environmental expenditure on the overall development of international tourism, coupled with a factor that may have been implied throughout the history of tourism but has not been properly analyzed, namely, happiness. If governmental expenditure plays a significant role in tourism, it will be considered an important factor in the future development of the tourism industry.

Following this Introduction, the next section is dedicated to literature on the relation between the environment, health, well-being and tourism. Section 3 analyzes the data, together with detailing the methodology of the study, while Section 4 details the calculations. Section 5 continues with the empirical results, as well as with the discussions. In the final part, we elaborate on several conclusions derived from this study’s results, together with policy recommendations and the main limitations of the study.

## 2. Literature Review

Researchers’ interest in describing the relationships between the environment, physical health and happiness, on one side, and tourism and travel on the other, stems from multiple reasons. First, it is a genuine interest in the means by which political and economic efforts for a cleaner environment, a better-performing healthcare system and an improved state of general well-being, can influence, and are influenced by, open-air activities, holidays and travel. Secondly, researchers and policy makers want to understand how environmentally friendly behaviors influence individuals’ subjective well-being and how these emotional states and attitudes can be used to promote more sustainable [5] and more socially responsible behaviors [6,7]. Just as important is that healthy individuals seeking well-being in a natural environment can be seen as more available for tourism and travel, as visitors or hosts; in this case, public authorities would be inclined to support tourism activity for economic and social outcomes.

In the following paragraphs, we will discuss the available literature on these bilateral or multilateral relationships between environmental quality and protection, happiness and, finally, travel and tourism.

### 2.1. Environmental Protection, Health, Well-Being and Happiness

People around the world recognize the importance of the natural environment and its protection for their continued health and well-being, and are increasingly aware of the particular risks and threats posed by climate change and public health crises in contemporary times.

The importance of the natural environment for people’s health and well-being is confirmed by numerous surveys at national, regional and international levels, such as the Eurobarometer surveys on public attitudes toward the environment [8], the Better Life Index surveys of the OECD [9] and the Health and Environment Strategy of the World Bank [10]. A growing number of data sets, based on existing or proposed indicators, including indicators of subjective well-being, show how people’s feelings and judgments about their quality of life depend on environmental factors such as geography and natural capital [11], temperature and precipitation [12], and air and noise pollution [13,14,15,16].

There is ample evidence in medicine, psychology and other related fields to suggest that travel and exposure to green and natural environments improves mental well-being, reduces stress, generates positive emotions and stimulates self-regulation and nervous balance [16,17,18,19,20]. Green and natural environments predispose people to activities, physical exercise and social interactions, improving mental or physical health, optimism and longevity; these aspects are therefore associated with happiness. Green environments are considered to have a lower degree of air and noise pollution, they positively influence people’s general state (physical and mental) through picturesque (”therapeutical”) landscapes [21], nature walks and discovery, and peace and calm, with direct and indirect effects on happiness [5,22].

In analyzing the relationship between the quality of the natural environment and well-being levels across OECD countries, the World Happiness Report of 2020 [23] finds significant effects of pollution in general on the degradation of happiness, and that, reciprocally, both green and blue spaces positively influence self-reported happiness. Surprisingly, the authors cannot strongly assert a clear negative effect of air pollution on happiness, despite the increasingly well-documented damage it inflicts on physical health. The complete causal impact of the natural environment on happiness is complex and occurs through different pathways and mechanisms [5].

There are a considerable number of studies and articles debating the effect of wealth accumulation (personal and national) on environmental concerns, but opinions fail to reach a consensus, at least relatively. Some studies examine individual-level correlations between living standards and environmental concerns, while others focus on the relationship between prosperity, happiness and (personal) care for a healthier, cleaner and more diverse environment. The importance of understanding the relationship between the quality (or concern for quality) of the environment and happiness has been a relatively new but fertile theme in research over the last decade. Some researchers suggest that the pursuit of happiness can also lead to concerns for the quality of the environment (including pressure on authorities to invest in environmental protection); however, finding a consistent relationship between the degree of happiness and the level of individual social or political involvement in environmental protection measures is difficult and relatively rare in the research of recent decades. The study of Sulemana [24] empirically investigated the effect of happiness on environmental concern in a sample of 18 countries and found that happier people are more willing to reduce their wealth (income) to protect the environment, and the finding holds for both residents of developed countries and those in less developed countries. Zhao and Sun [22] examined the effect of environmental performance on subjective well-being under different contexts and levels of economic development in China, and found that public satisfaction with environmental performance will significantly increase their happiness. They found that Gross Domestic Product (GDP) moderates this effect, stating that people have high expectations of happiness in provinces that have high GDP. MacKerron and Mourato [12] addressed the relationship between momentary subjective well-being and individuals’ immediate environment within the UK. On average, the study participants reported being significantly and overall happier outdoors in all types of green or natural habitats than in urban environments. The study of Song et al. [16] shows that the subjective perception of air pollution has significant negative effects on respondents’ happiness; this is more pronounced in people with either health conditions, or who are middle-aged and elderly. Meanwhile, Lin et al. [25] analyzed, in greater detail, the health risks caused by fine particulate matter and other air pollutants on residents and visitors, with a focus on urban areas of high touristic significance.

Modern societies are considered to have failed, from a relational and environmental perspective, in providing a link between environmental degradation and well-being. The economic growth and prosperity of members of rich societies is overshadowed by their dissatisfaction with increasing pollution, environmental degradation and loss of biodiversity. In other words, economic growth does not necessarily lead to happiness, while economic affluence is seen as “compensation for the emotional distress and loss of resources caused by scarce social and affective relationships” [26]. Citizens living in the richest countries are not always the happiest [27,28], especially if they note the degradation of the natural and social environment [29], or the increase in insecurity and volatility [30]. Implicitly, the question arises about whether public spending for environmental protection, by improving social quality of life, could also improve the level of happiness in the long run? Trying to answer this question, D’Uva et al. [6] state that “public spending on environmental protection responds to a worsening in the quality of life caused by overexploitation of natural resources and aims to restore happiness by providing a more sustainable community development”. Furthermore, the quality of social life, together with an increase in the level of happiness, are the result of governments spending on environmental protection. However, this spending is coupled with a direct long-term equilibrium that manifests at the level of happiness and environmental expenditure.

### 2.2. Perspectives on Happiness, Environment and Tourism

#### 2.2.1. Happiness, Environment and Tourism: A Tourists’ Perspective

We therefore found that, beyond the material aspects, the happiness of individuals also depends on other factors [31], such as values and ideology, social relations, the environment, proximity to nature, etc. In the latter case, tourism, travel and physical activities in nature can improve people’s psychological state, involve individuals in protecting the physical and social environment [32] and contribute to people’s well-being and happiness [33]. Lin et al. has shown that, among the elderly, specific forms of tourism, such as cultural or religious tourism, “could construct a sustainable and friendly life and leisure environment to promote physical and mental health” [34].

Filep and Deery [35] argue that, in contrast to philosophy and the social sciences, the topic of happiness is surprisingly under-explored in tourism studies, although tourism is perceived as more than a break from daily routines—an activity of health and well-being. The understanding of happiness in relation to tourism activities needs to be reinterpreted, and then, evaluated in the main phases of the travel experience—anticipatory, on-site and subsequent (reflective). They conclude that tourist happiness is a state in which the tourist experiences positive emotions (joy, interest, contentment and love) and is involved in positive experiences. Happiness is associated with holiday activities, an important image that sheds light on the personal value and quality of tourism experiences for individuals.

For Corvo [36], the problems of Western society alienate the individual, subject them to crises and difficulties in interpersonal relationships and cause them suffering and fragility, reducing expectations and hopes for the future. The individual is insecure and dissatisfied, looks forward to holidays and vacations and projects on them great expectations and hopes. Indeed, tourists prefer places with a strong connection with nature, with outdoor activities, and they want to enjoy the beauty of the landscape. However, in practice, holidays and vacations do not always turn into perfect experiences, because it is difficult to escape the routine of everyday life. In addition, the mechanisms of tourist facilities pull the individual back into the spiral of consumerism. Difficulties in feeling happy through tourism depend on the individual and their inability to separate frustrating daily life from leisure.

Cheng and Li [37] examine tourist happiness by linking it to a specific destination, where happiness is shaped by the destination image, service quality, tourist behavior at the destination and life satisfaction. They believe that life satisfaction can predict, to a great extent, the “eudaimonia” (a Greek word for the state or condition of ‘good spirit’), and the positive and negative effects related to the tourism experience. However, tourists are often reluctant to relate their travel experiences to the negative effect.

Nawjin [38] aimed to find out if vacationing improves tourist happiness in the long run and found that tourists on vacation were marginally happier than those not on vacation, and that there is a small causal effect of holidays on good mood, but not bi-directional (i.e., happiness/well-being/optimism do not predispose people to going on holiday). Additionally, those who went on vacation appeared to be marginally happier than those who did not. However, the effect of holiday travel on happiness is short-lived and unrelated to the size and frequency of such travels. Whereas life satisfaction and good mood experience a continuous decline, Kwon and Lee [39] found that they improve before the trip and last about a month after the trip. The authors argue for increasing the role of satisfaction and positive travel experience in improving mental health and prolonging happiness.

Bimonte and Faralla [32] point out that travel and vacations in nature can boost emotional and physical well-being and community spirit, temper materialistic behaviors and diminish pressure for social success. They are also useful for the protection and conservation of the physical and social environment and can boost emotional and physical personal well-being.

Pinna et al. [40] point out that the happiness effects associated with vacations and travel are also conditioned by the moral values of individuals, their psychological or emotional profile and their socio-demographic and situational characteristics and, implicitly, they can transcend cultural factors. Mccabe and Johnson [41] address the social tourism side and consider changes in well-being among low-income people who have received financial support to access a holiday (so-called “social tourists”). They find that tourism contributes to well-being (psychological, relaxation and family life), but those social tourists also have inferior levels of subjective well-being compared to the general population.

Ram et al. [7] assert that current models of mobility in tourism are not sustainable, although the desired sustainable conduct should increase the length of stay, use of public transportation and use of short-distance destinations. Instead, tourists prefer speed, individualism, long distances and short but frequent stays. More precisely, “happiness, travel motivations and perception of distance set barriers for desirable behavioral change”, that is, vacations also imprint unsustainable behaviors that are different from every-day, ecological behaviors. Effective policies are recommended to break the “speed-distance-demand loop” and the recognition of the role of happiness in sustainable tourism strategies. Authors such as Liu and Suk and Geng et al. [42,43] sought to understand the intrinsic relationships between environmental quality and tourism and the coupling and coordination between the tourism economy and the environment. The results of these studies can help us to evaluate, compare and predict the trends that characterize this relationship, as well as define local and regional policies for the sustainable development of tourism and the economy in general.

#### 2.2.2. Happiness, Environment and Tourism: A Residents’ Perspective

Researchers agree that for developing states, but especially for small island states, tourism is a chance for and a path to economic growth and modernization, a fact that has led to the expansion of the tourism sector in these states for more than five decades. However, the links between tourism development and the economic, social and personal well-being of residents are ambiguous at best. Pratt et al. [44] question whether, in these contexts, tourism makes residents happy? After careful analysis of two localities in Fiji, they conclude that although residents of tourism-dependent settlements are relatively materially more prosperous, those in localities with very few links to tourism appear to be happier in many aspects of daily life.

Also exploring a small island destination, Rivera et al. [45] bring new perspectives on how residents evaluate their own happiness; this is not necessarily related only to material well-being, but introduces other factors such as quality of life and comparisons of life situations. They show that the tourism–resident happiness relationship correlation is positive but slim and not exclusive, and non-income factors (such as social comparisons) have a large impact in defining happiness for residents in these places.

The residents’ negative perceptions towards the economic, social and environmental impacts of tourism are the subject of Sánchez-Teba et al. [46], who conducted a study in a tourist town in Spain. They also add residents’ loyalty to their city to the analysis and find that negative views on tourism reinforce each other and affect the residents’ happiness. Instead, loyalty (perception of prestige and pride) to the living place (neighborhood) has a positive and direct impact on happiness, i.e., an increase in loyalty is associated with an increase in happiness.

Okulicz-Kozaryn and Strzelecka [47], using European Social Survey data, consider that domestic tourists contribute more to the happiness of locals than foreign tourists, and tourism in regions with low levels of development contributes more to happiness than tourism in destinations with high levels of development. Subsequently, the development of conventional tourism can contribute to overall development and has positive effects on an the area/destination, up to a point. Very popular destinations experience a non-existent and even negative relationship between tourism and happiness, i.e., more tourism probably makes residents more unhappy and they, in turn, have more negative attitudes towards tourism, affecting tourists’ satisfaction [48].

It is a well-known fact that tourism facilitates development; however, there is a warning, underlined by various studies and organizations, that emphasizes the need to have the host communities at the core of all processes that involve destination planning [49]. At the same time, the indicators tracked in surveys regarding the happiness index of the host populations must be contextually adapted [50]. Research and actions are needed to avoid the phenomena of over-tourism and pushback from locals disappointed and affected by tourism pressure, and to enable host communities and wider stakeholders to guide tourism development towards happiness, well-being and sustainability [49].

Other researchers have promoted a more pragmatic and positive view, investigating whether the happiness of some (host) nations can be considered an element of attraction for international tourists, similar to tangible (sites, architectural ensembles and monuments) or intangible heritage (such as traditions, customs and events), as long as the search for experiences (emotional, skills or gastronomic) can be an important reason in the decision of choosing vacations and trips.

Of course, happiness, or rather, the lack of it, a state of dissatisfaction—not only material, but also social or personal—among the population of a country. The perception of authorities’ disinterest in the environment or public health leads to higher emigration rates [5,6,31,51,52], the weakening of social cohesion and mediocre economic performance. However, here, we are interested in the short-term effect and in a specific sector, i.e., travel and tourism, namely, does national happiness attract more arrivals and receipts from international tourism?

The authorities and the tourism industry recognized this a long time ago and have been running promotional campaigns for years in which they present happy tourists and locals, but at the level of scientific research, things are more complex. Thus, most studies on this topic start from the assumption that international tourists are interested in “happy” destinations, because, according to the Aristotelian tradition, individuals have a fundamental preference for exposure to happiness [51].

If this is true, then “happier” countries should pay attention to the state of happiness of the population and use it as an intangible asset, “selling” it to international visitors. The fact that “happier” countries attract more tourists, and that they (tourists) spend more money there, seems to be widely accepted in theory and practice, but the question is: How much of this spending is generated by the influence of local happiness on “happiness-sensitive” tourists [51]? Of course, research must focus on identifying the personal characteristics, demographics, levels of happiness and consumption habits of the market segments most influenced by considerations of happiness, and on understanding which factors most influence tourists’ perception of the happiness of destination countries.

### 2.3. International Tourism and Global Public Health

The continuous expansion of international tourism in recent decades (interrupted but not stopped by the recent pandemic, economic and political crises) has also raised other challenges, especially in relation to the risks to public health. The increase in international travel and migration can interfere with public health both in the destination country and at home; infectious diseases are more common after mass gatherings, business and touristic trips or relaxing activities away from home, and also during flights or other means of public transportation [53] and during pilgrimage or other religious and cultural events [54].

Analyzing international tourism and its global public health consequences, Richter [55] shows that there is a public health crisis (that is unique and growing) associated with global tourism, threatening not only tourists, but also the host societies and nations of origin of the tourists. Regulation and coordination erode as the need for international cooperation increases. The health and safety challenges posed by globalization (in general) and international tourism (in particular), respectively, call for stronger coordination, political will, surveillance and planning, in other words, tourism can play a role in increasing the awareness of global health crises and, subsequently, aid in combating them [56], which is referred to as ”pandemic prevention via tourism” by Jiang et al. [57]. Postponement, inaction, denial and the search for immediate satisfaction will make late options less pleasant and more expensive (human and economic).

An economic perspective, based on the data generated by the effects of the COVID-19 pandemic, especially on international tourism, is brought by Salisu et al. [58]. They point out that the outbreak of the COVID-19 pandemic has led to an unprecedented crisis in global health and the financial market, the tourism sector being among the major victims with an almost total collapse due to economic blockages and traffic restrictions. Meanwhile, the health sector has experienced a considerable boom. For Rossello et al. [59], in some countries, favorable climate conditions for tourism are often associated with favorable conditions for infectious diseases, with developmental constraints, which are proving more acute in poor countries, dependent on the economic contribution of tourism to the national economy. The eradication of infectious diseases (malaria, dengue, yellow fever and Ebola) in the affected countries would result in a significant increase in arrivals and revenue in international tourism for these countries, and for others.

Despite a rich body of literature, most studies investigate a bivariate relationship between tourism and either health, environmental quality or well-being. Our paper attempts to describe a multivariate relationship between the above-mentioned variables, taking into account interactions between healthcare and environmental expenditure and happiness in international tourism (arrivals and receipts).

## 3. Data and Methodology

This study aims to examine the long-term and the short-term relationships between international tourism, measured in number of arrivals (ARRIV) and receipts (REC), and three different factors: happiness (HAPP), environmental expenditure (ENV) and health expenditure (HEALTH) in the 27 European Union (EU) countries. In order to investigate the relationship between these variables inside the EU27, we used panel data analysis. Given the limited availability and accessibility of the data for environmental expenditure, healthcare expenditure and variable happiness in some countries, this study used data from 2000 to 2019 and a balanced panel of EU27 countries. In Table 1 we present the names of the analyzed variables used in the study, the definitions of the variables, and the data sources.

Before transforming the data, in the case of environmental expenditure, we converted currency from EUR into USD, in order to have the same currency for all the variables. The descriptive statistics for the five analyzed indicators are presented in Table 2.

Since each variable is expressed using a different unit of measure (i.e., number of tourists, million EUR, current international USD and percent), before performing any analysis, the variables’ data must be standardized or normalized [63]. In this study, all the variables were transformed to their natural logarithmic forms, since natural logarithms smooth all the data used for analysis.

We chose EU27 data because most European Union countries have a long tradition of environmental and healthcare policies, the tourism sector is likewise well-developed and representative and statistical data are easily accessible, harmonized across member states and available for the entire analyzed period. In particular, the present paper builds on our previous research on the relationship between environmental quality and tourism development, and between economic growth, pollution and health in EU countries [64,65,66].

Starting from the literature review and the research questions of the present study, we further propose testing the following hypotheses at the EU27 level:

**H1.**  *There is a long-term effect of happiness, environmental expenditure and health expenditure on the international tourism.*

**H2.**  *There is a short-term effect of happiness, environmental expenditure and health expenditure on the international tourism.*

## 4. Calculation

In order to fit the panel data model [67], an important step is to test the cross-section dependence. This type of correlation can result from common global shocks that can have different effects from one country to another, and the impact of cross-sectional dependence on estimation depends on a variety of factors. Thus, if T (time-series dimension) is greater than N (cross-sectional dimension), the Lagrange multiplier (LM) test [68] will be used. Taking into account the limits of the Breusch and Pagan LM test when N (cross-sectional dimension) is large, Pesaran [69] proposes another coefficient. Thus, when T is small and N is large, which is the most commonly encountered situation in panels, we can used Pesaran’s [69] cross-sectional dependence (CD) test and the equation is:

(1) $$\mathrm{CD}=\sqrt{\frac{2T}{N(N-1)}}\left(\sum_{i=1}^{N-1}\sum_{j=i+1}^{N}\left(\rho_{ij}\right)\right)$$

where N represents the cross-sectional dimension, T represents the time period and p_ij_ represents the pair-wise correlation coefficients of the residuals. The null hypothesis of this test is no cross-sectional dependence and the alternative hypothesis is cross-sectional dependence (correlation). Because in our study, T < N, we used the cross-sectional dependence (CD) test suggested by Pesaran [69].

Using the CD test proposed by Pesaran [69], in Table 3, we present the results of the cross-sectional dependence analysis. The results obtained after the application of this test show that the null hypothesis (no cross-sectional dependence) is rejected and the alternative hypothesis regarding cross-sectional dependence is accepted at a significance level of 1%.

Given that the cross-sectional dependence is confirmed, we continue the empirical analysis with the determination of the series stationarity. To test the stationarity of the analyzed series, we use four different types of panel root unit tests, which are superior to time-series unit root tests. Thus, in our study we use Levin, Lin and Chu (LLC) [70]; Im, Pesaran and Shin W-stat (IPS) [71]; ADF–Fisher Chi-square [72]; and PP–Fisher Chi-square [73]. In the case of the LLC test, the null hypothesis shows a unit root and assumes a common unit root process, while the null hypothesis of the other three tests shows a unit root and assumes an individual unit root process. Cointegration can only be used in a situation where the series are stationary at the same level. However, the ARDL (Autoregressive Distributed Lag) test allows for the study of cointegration in a situation where a certain series is stationary at the level and another series is stationary at the first difference level. Therefore, this test can only be used if the series are integrated (I(0) and I(1)) or there is a mixture of both [74,75].

The results presented in Table 4 show that the variables LnARRIV, LnREC, LnENV and LnHEALTH are stationary (at the 0.01 level) at the first difference, while the variable happiness (HAPP), is stationary at its level, at the 0.01 level. Thus, we can argue that the data are either I(0) or I(1), with this aspect giving the opportunity to estimate the short-term and long-term relationships between the variables. Additionally, because the analyzed variables are stationary at the level or at their first differences levels, cointegration analysis can be performed in this case. Thus, we proceed with the study of cointegration by using the Pedroni–Johansen cointegration test. Pedroni [76] proposed a panel tests based on within- and between-dimension approaches. The within-dimension approach includes [76] “panel v-Statistic, panel rho-Statistic, panel PP-Statistic and panel ADF-statistics”, while the between dimension approach includes: “group rho-Statistic, group PP-Statistic and group ADF-statistics”. All the statistics presented above are based on an average value of the individual autoregressive unit root tests of the individual members of the panel data set [75,76,77].

According to the results, we can observe that in the case of the first two estimated models, for three out of four cointegration tests (panel PP-statistic and panel ADF-statistic) the null hypothesis shows that there is a cointegration between the studied variables, while in the case of the last two models, for two out of four tests (panel PP-statistic and panel ADF-statistic) the null hypothesis shows that there is cointegration between the analyzed variables. Therefore, we can state that there is a long-term relationship between international tourism, happiness and environmental expenditure, respectively there is a long-term relationship between international tourism, happiness and healthcare expenditure. Additionally, in the case of the last three statistics (between-dimension approach), the null hypothesis is rejected for two out of three tests (PP-statistic group and ADF-statistic group). Pedroni [77] suggests that, among the results of these statistics, we can identify contradicting results. However, Pedroni [77] reported that if T is less than 100, the group and panel ADF statistics have the best power properties, while in the case of the panel v and group rho statistics, the performance is comparatively poorer. Moreover, ADF statistics perform better if the errors follow an autoregressive process [78]. Finally, considering the results presented in Table 5 and the above-mentioned limitations, we can say that the analyzed variables are cointegrated.

Given that the analyzed data are either I(0) or I(1), we can estimate the short- and long-term relationships between the variables by using the ARDL model in the error-correction form. We estimate the model starting from the Pooled Mean Group (PMG) developed by Pesaran et al. [75]. The ARDL model is:

(2) $$Y_{it}=\sum_{j=1}^{p}\alpha_{ij}y_{i,}{}_{t-j}+\sum_{j=0}^{q}\beta_{ij}X_{i}{}_{,t-j}+\mu_{i}+\varepsilon_{it}$$

where i represents the number of groups (i = 1, 2, 3, …, N), t represents the time (t = 1, 2, 3, …, T), X_i,t-j_ is a K x 1 vector of explanatory variables for group i, and μ_i_ represents the group-specific effect [75].

A cointegration relationship between variables is their response to a long-term equilibrium relationship. Thus, Equation (2) can be reparametrized [79] to yield an error-correction (ECM) equation:

(3) $$\Delta y_{it}=\delta_{i}\left(y_{i,t-1}-\beta_{i}X_{i,t}\right)+\sum_{j=1}^{p-1}\gamma_{i,j}^{}\Delta y_{i}{}_{,t-j}+\sum_{j=0}^{q-1}\lambda_{i,j}^{}\Delta X_{i}{}_{,t-j}+\mu_{i}+\varepsilon_{it}$$

where y_it_ represents the international tourism (number of arrivals (ARRIV) and receipts (REC)) for country i at time t, X_i,t_ represents the proxies for happiness (HAPP), environmental expenditure (ENV) and health expenditure (HEALTH) for country i at time t, and ɣ_i,j_ and λ_i,j_ represent the short-term coefficients. The parameter δ_i_ is the error-correcting speed of adjustment and β_i_ is the long-term parameter that shows the relationship between y and X. The short-term effects are measured by λ_i,j_ and represent the coefficient associated with the ΔX variables.

Thus, the PMG estimator allows for the determination of the constant and the short-term coefficients, and also allows for the determination of the long-term coefficients. An important requirement for the consistency of this procedure is that there is a long-term equilibrium relationship between the analyzed variables, and this can be determined with the help of the error-correction parameter, which must be negative and statistically significant.

## 5. Empirical Results and Discussion

The long-term elasticities and the short-term elasticities are estimated using the ARDL panel method for all 4 models. The two models presented in Table 6 and Table 7 have the same form as Equation (2). In the proposed models, in addition to determining the impact that the independent variables have on international tourism, we also included a product variable that will capture the interaction between the independent variables. Thus, in order to determine how variation in environmental expenditure (LnENV) produces changes in happiness (thus having an effect on international tourism) and how variation in happiness produces changes in environmental expenditure’s effect on international tourism, we have included in the model a product variable [80], LnHAPP* lnENV. Additionally, in order to detect how variation in healthcare expenditure produces changes in happiness (thus having an effect on international tourism) and how variation in happiness produces changes in healthcare expenditure’s effect on international tourism, we have included the product-variable LnHAPP* lnHEALTH.

Regarding the long-term relationship presented in Table 6, we can state that between international tourism and the number of arrivals, happiness and environmental expenditure in the case of the first model, and between international tourism and the number of arrivals, happiness and healthcare expenditure in the case of the second model, there is a possible long-term relationship considering that the ECT coefficient is statistically significant at the 0.01 level and it is also negative.

If we analyze the long-term effect of the exogeneous variables on the endogenous variable, ARRIV, we can state that the results of the variable happiness show a significant and a positive long-term effect on the international number of arrivals in the 27 European Union countries in both models. Therefore, the 1% increase in happiness can support between 6% and 9% of the international number of tourists in the long run. According to the results obtained at the sample level, we can affirm that in the long run, considering the high values of the coefficients, happiness represents an important factor in the 27 EU countries analyzed in terms of the levels of and increases in the international number of arrivals.

According to the results presented in Table 6, in the long run, environmental expenditure has a positive and statistically significant effect (at the 0.01 level) on the number of international tourist arrivals in all 27 EU countries. Thus, long-term estimates have revealed that environmental expenditure causes an increase in the number of international tourist arrivals. Thus, at the level of our sample, the environmental expenditure can support 2.5% of the number of international tourist arrivals in the long run. According to the results, environmental conditions increase international tourism, but at small levels compared to happiness. Thus, we could consider that the countries that invest in the protection and development of the environment, especially in the long run, create an opportune context for the increase in the number of international tourists.

If we analyzed the effect of health expenditure on the number of international tourist arrivals, we would have a 1% increase in health expenditure and this could lead to an increase of 7.28% of the number of international tourist arrivals in the long run. Therefore, considering the results obtained at the sample level, we can state that both environmental expenditure and health expenditure have a positive long-term effect on international tourism in the panel. Moreover, health expenditure influences the number of international tourists to a greater extent than environmental expenditure, by almost 1%. Thus, the results confirm the first hypothesis (H1) of this study in the case of international tourist arrivals.

In our equation, the coefficient of the product term describes how the causal effect of one exogenous variable on the endogenous variable is affected by the variation in the other exogenous variable [81]. Therefore, in order to identify the effect of the interaction between happiness and environmental expenditure on the number of international tourism arrivals with the help of the product term, we take into account the fact that the coefficient of this term describes both the way in which the variation in environmental expenditure produces changes in happiness—having an impact on international tourism—and how variation in happiness produces changes in the effect of environmental expenditure on international tourism. The results of the first model show that the coefficient of the product term (LnHAPP* lnENV) is negative and the coefficients of the individual variables, LnENV and LnHAPP, are positive. Thus, a 1% increase in environmental expenditure could lead to an increase of 2.50% of the number of international tourist arrivals when happiness is not considered. Nevertheless, variation in happiness produces changes in environmental expenditure with an effect on the number of international tourist arrivals; thus, a 1% increase in happiness leads to a decrease of 0.99% in changes in environmental expenditure and their effect on the number of international tourist arrivals. Therefore, at the panel level, the results show that if the level of the residents’ happiness is not taken into consideration, the number of international tourists would be influenced by environmental expenditure to a higher extent than when happiness is taken into consideration. It follows that the effect of environmental expenditure on the number of tourists decreases when we take into account a rise in the happiness levels of the residents. We can assume that a higher degree of happiness among the residents would lead to a higher level of protection of the environment. However, environmental expenditure does not decrease, and international tourists would rather evaluate the level of residents’ happiness than these expenditures. In the second model, which includes health expenditure, we have a similar result. Thus, a 1% increase in health expenditure could lead to an increase of 7.28% in regard to the number of international tourist arrivals when happiness is not considered. If we study how variation in happiness produces changes in the effects of health expenditure on the number of international tourist arrivals, we can state that a 1% increase in happiness leads to a decrease of 3.75% in changes in health expenditure and their effect on the number of international tourist arrivals. If the level of happiness is growing, there will be a diminishing impact of the health expenditure of the residents on the number of international tourists. Therefore, if the residents’ happiness level was increased, healthcare expenditure, allocated by governments, would have a lower impact on the number of tourists. These would be influenced to a greater degree by the level of happiness, and less by the sums allocated by the government for environmental expenditure.

Regarding the short-term effect of environmental expenditure and health expenditure, respectively, we can affirm that these two variables do not influence the number of international tourist arrivals in the panel. According to the results presented in Table 5, the short-term coefficients are not statistically significant, and the p-values are higher than 0.05. Therefore, at the level of EU27 countries, the second hypothesis (H2) of this study cannot be confirmed in the case of international tourist arrivals.

The analysis of the cross-section results in the short run have revealed the same situation to the one presented at the panel level in the case of almost all the countries analyzed. According to the results presented in Table A1, in the case of France, Greece, Lithuania and Romania, environmental expenditure and health expenditure have a significant impact on the number of international tourist arrivals in the short run, while in the other countries, these two variables do not have a statistically significant influence on the number of international tourist arrivals. Additionally, in the case of happiness, in the short run, this indicator does not have a statistically significant influence on the number of international tourist arrivals, the result of which is similar to that obtained at the data panel level. The long-term results for each country show that in countries such as Cyprus, the Czech Republic, Croatia, Estonia, Greece, Italy, Ireland, Lithuania, Finland, Luxembourg, Latvia, Slovakia, Slovenia, Poland, Portugal, Spain and Sweden, there is a long-term relationship between international tourist arrivals, happiness and environmental expenditure, while in countries such as Austria, Belgium, Bulgaria, Denmark, France, Germany, Hungary, Malta, the Netherlands and Romania, we did not identify a long-term relationship between these variables. We also studied the long-term relationship between international tourist arrivals, happiness and health expenditure. We identified a significant long-term relationship in the case of Bulgaria, the Czech Republic, Croatia, Estonia, Finland, Greece, Hungary, Lithuania, Luxembourg, Latvia, Poland, Portugal, Romania, Slovakia, Spain and Sweden, while in Austria, Belgium, Cyprus, Denmark, France, Germany, Ireland, Italy, Malta, the Netherlands and Slovenia, we did not identify a long-term relationship between these variables.

According to the results presented in Table 7, we can argue that in the case of models 3 and 4, where the endogenous variable is represented by the variable tourism receipts, the effects of the exogenous variables on tourism receipts are approximately similar to those obtained in models 1 and 2. Thus, happiness has the greatest impact on tourism receipts, while environmental expenditure and health expenditure influence tourism receipts to a lower degree. In the long run, a 1% increase in happiness can support between 4% and 9% of tourism receipts, while a 1% increase in LnENV leads to an increase of 2.63% in tourism receipts. Regarding the effect of health expenditure on tourism receipts, the long-term estimations revealed that health expenditure causes an increase in tourism receipts in the panel countries, and the health expenditure coefficient is positive and statistically significant at the 1% level. Therefore, at the level of our sample, health expenditure could lead to an increase of 2.01% in tourism receipts in the long run. According to the results, the environmental conditions increase tourism receipts to a greater extent than health and to a lower extent than happiness. Thus, according to the results, the first hypothesis (H1) of this study is also confirmed in the case of receipts from tourism.

In these two models (model 3 and model 4), we include the product term. In model 3, the product term coefficient between happiness and environmental expenditure is negative, while the coefficients of these two variables are positive. Thus, a 1% increase in environmental expenditure can support 2.63% of tourism receipts when happiness is not considered. A variation in happiness produces changes in environmental expenditure with an effect on tourism receipts; thus, a 1% increase in happiness leads to a decrease of 1.03% in changes in environmental expenditure and their effect on tourism receipts. In the fourth model, according to the results, a 1% increase in health expenditure can support 2.01% in regard to tourism receipts when happiness is not considered. If we study how variation in happiness produces changes in the effects of health expenditure on tourism receipts, we can state that a 1% increase in happiness leads to a decrease of 0.66% in changes in health expenditure and their effect on tourism receipts. In this case, it can be observed that an increase in the level of happiness of the residents modifies the effect of healthcare expenditures on tourism receipts to a higher degree than environmental expenditure. Therefore, when the level of happiness of the residents is higher, international tourists are more preoccupied by the healthcare expenditure allocated by the country’s government, and less by environmental expenditure.

In the short run, we did not find a statistically significant influence of environmental expenditure on tourism receipts, while in the case of health expenditure, we identified a negative impact on tourism receipts. Therefore, we can argue that at the level of EU27 countries, the second hypothesis (H2) is partially verified in the case of tourism receipts. The cross-section results in the short run show, in the case of almost all the analyzed countries, the same relationship between the variables to that presented at the panel level. Thus, the results presented in Table A2, show that only in the case of Lithuania does health expenditure have a significant impact on tourism receipts in the short run, while in the other 26 countries, the independent variables do not have a significant influence on tourism receipts. The long-term results for each country show that in countries such as the Czech Republic, Denmark, Finland, France, Germany, Ireland, Italy, Luxembourg, Latvia, Malta, the Netherlands, Poland, Slovakia, Slovenia, Spain and Sweden, there is a long-term relationship between tourism receipts, happiness and environmental expenditure, while in countries such as Austria, Belgium, Bulgaria, Cyprus, Croatia, Estonia, Greece, Hungary, Lithuania, Portugal and Romania, we did not identify a long-term relationship between these variables. Regarding the long-term relationship between tourism receipts, happiness and health expenditure, we identified a significant relationship in the case of Belgium, Bulgaria, Cyprus, the Czech Republic, Croatia, Estonia, France, Greece, Germany, Hungary, Italy, Lithuania, Luxembourg, Latvia, Malta, The Netherlands, Poland, Portugal, Romania, Slovakia, Spain and Sweden, while in Austria, Finland, Denmark and Ireland, we did not identify a long-term relationship between these variables.

In order to capture the direction of the relationship between international tourism (ARRIV and REC), happiness, environmental expenditure and health expenditure, we used the panel causality tests built by Dumitrescu and Hurlin [82].

**Figure 1:**
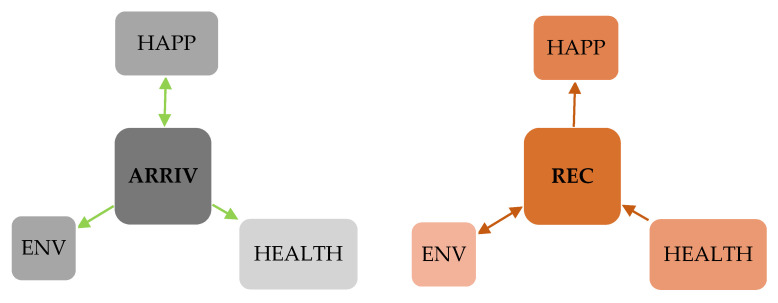
Results for Dumitrescu and Hurlin panel causality test. Source: own computations using EViews 12.

Starting from the results of the Dumitrescu and Hurlin test, we outline the results according to Figure 1. The number of international tourist arrivals is a cause for happiness, as well as for an increase in the two expenditures for environment and health. Thus, in the case of the two expenditures analyzed, we identified a unidirectional causal relationship from the number of international tourist arrivals, and in the case of happiness, we identified a bidirectional causal relationship. In the case of tourism receipts, we identified a bidirectional causal relationship between tourism receipts and environmental expenditure, and a unidirectional causal relationship from health expenditure to tourism receipts and, respectively, from tourism receipts to happiness.

## 6. Conclusions

Tourism is a highly sensitive and flexible industry. It is affected by social, political, economic and environmental issues, stemming from poverty to political instability, economic crises and various natural disasters and pandemics. Despite all these grievous issues, tourism is not abandoned as an industry. It forges on and adapts to new contexts, to various new rules and regulations, and it transforms constantly not only to benefit the industry, but also to favor and develop the regions where it operates. In this context, tourism favors human communities and the environment, by protecting them and by increasing health, well-being and happiness. Together with legislators and various state institutions, the tourism industry aims to be not only profitable, but also sustainable, even at the cost of making important and costly changes to its operations.

In this article, we analyzed the long-term and the short-term relationships between international tourism (measured by the number of tourist arrivals and by tourism receipts) and happiness, environmental expenditure and healthcare expenditure on a panel of the 27 European Union countries in the 2000–2019 time period, employing a panel ARDL model. The first step in our analysis was to normalize the data using the natural logarithm. After the data were normalized, we tested for cointegration using the Pedroni test, which showed the presence of cointegration. The results of the panel root unit tests show that the series of the variable HAPP is stationary at the level (significance threshold of 5%), while the series of variables ARRIV, REC, ENV and HEALTH are stationary only at the first difference level.

Since the order of integration of these series is either I (0) or I (1), we used ARDL to determine both the short- and long-term relationships. Our research shows that happiness has the greatest impact on international tourism, both on the number of arrivals and on tourism receipts, but only in the long run. Therefore, a 1% increase in happiness can support an average increase of 8% in international tourist arrivals in the long run, and an average increase of 6% in tourism receipts in the long run. According to the results in the short run, at the panel level, happiness does not influence international tourism. Regarding environmental expenditure, we found that it determines an increase in international tourism in the long run, i.e., a 1% increase in environmental expenditure, can support an increase of 2% in international tourism. Additionally, healthcare expenditure influences the number of arrivals, at the panel level, to a greater extent than environmental expenditure.

Moreover, variation in happiness influences the effects of both environmental expenditure and health expenditure on the number of international tourist arrivals. A 1% increase in happiness leads to a decrease of 0.99% in changes in environmental expenditure and of 3.75% in changes in health expenditure and their effect on the number of international tourist arrivals. In the case of receipts, the impact is reversed. Thus, variation in happiness produces changes in the effects of environmental expenditure and health expenditure on tourism receipts. A 1% increase in happiness leads to a decrease of 1.03% in changes in environmental expenditure and their effect on tourism receipts, and to a decrease of 0.66% in changes in health expenditure and their effect on tourism receipts.

Overall, the results of our research suggest that international tourists are more likely to travel to and spend more in countries that are happier and which invest more in the environment and public health. Moreover, if health, environmental quality and, most importantly, national happiness are seen responsibly by decision-makers and understood to be intangible assets influencing international tourism arrivals and revenue, they can form a basis for nationally significant strategies. These positive effects will not be limited to the tourism industry, destination areas and local and national budgets, but will spill over to the national economy and society as a whole, and most importantly, to the well-being of each individual.

The policy implications of this study imply the necessity for national legislators to create political and economic environments that enable and facilitate investments in environmental protection and sustainability. Creating laws and regulations must be in accordance with tangible objectives, therefore allowing the tourism industry to flourish and become more sustainable, and also to become a factor of prosperity for local communities. Since traveling is a proven means through which exposure to nature reduces stress, and increases positive emotions, well-being and nervous balance, policies should favor increased mobility and the development of national parks and protected areas, together with the increase in green spaces (parks and vegetation), even in highly congested regions. Happiness and well-being are natural outcomes that favor productivity, travel and expenditure for increasing health, thus creating and sustaining an economic environment that supports local communities and a better understanding of the values that keep human beings in healthy relationships, both regionally and globally.

In the present study, we encountered a number of limitations, specifically those related to obtaining a more comprehensive data set, in terms of the time period, that was large enough to be significant. Thus, considering the analyzed variables, the time period considered was reduced to 20 years. Moreover, future research aims to analyze more countries in Europe and could include a larger number of variables related to the environment, variables related to different types of health expenditure, variables that highlight the economic development of a country and variables that analyze domestic tourism. Another question is whether the results can be extrapolated beyond the EU, considering the great diversity of healthcare and environmental policies, as well as the variation in national happiness.

## Figures and Tables

**Table 1:** Definition of variables and data sources.

Variable Name	Variable Definition	Source
Dependent variables (natural logarithm)		
International tourism		
lnARRIV	International tourism measured— the number of arrivals	World Bank [60]
lnREC	International tourism receipts— expressed in current USD	World Bank [60]
Independent variables (natural logarithm)		
lnHAPP	Happiness Index—%	World Database of Happiness [61]
lnENV	Environmental expenditure— Million EUR	Eurostat [62]
lnHEALTH	Health expenditure per capita— purchasing power parities (PPP), current USD	World Bank [60]

**Table 2:** Descriptive statistics.

	International Tourism		HAPP	ENV	HEALTH
	ARRIV	REC			
	Number	current USD	%	current USD	current USD
Mean	27,739,341.85	14,513,790,133	6.35	3840.82	2454.55
Standard Dev.	41,238,119.11	33,790,174,067	1.00	6329.58	1854.42
Median	10,926,000	6,619,000,000	6.40	1055.81	1917.41
Minimum	805,000	153,000,000	3.80	−50.47	69.89
Maximum	211,998,000	689,900,000,000	8.60	29,187.37	7670.59
Skewness	2.73	15.10	−0.16	2.09	0.70
Kurtosis	7.87	296.77	−0.46	3.53	−0.71
Observation	540	540	540	540	540

Source: own computations using EViews 12.

**Table 3:** Cross-sectional dependence analysis.

	Pesaran CD Test	
Variables	Statistic	Prob
lnARRIV	62.88	0.0000
lnREC	71.05	0.0000
lnHAPP	28.59	0.0000
lnENV	69.16	0.0000
lnHEALTH	78.44	0.0000

Source: own computations using EViews 12.

**Table 4:** Panel unit root test.

	LnARRIV	LnREC	LnHAPP	LnENV	LnHEALTH
Levin, Lin and Chu t					
Level	−0.73 (0.23)	0.68 (0.75)	−4.28 (0.00)	2.21 (0.98)	4.47 (0.99)
First diff	−12.78 (0.00)	−13.68 (0.00)		−11.72 (0.00)	−9.61 (0.00)
Im, Pesaran and Shin W-stat					
Level	0.10 (0.54)	−1.22 (0.11)	−3.86 (0.00)	0.20 (0.58)	2.04 (0.97)
First diff	−10.54 (0.00)	−11.35 (0.00)		−10.75 (0.00)	−7.39 (0.00)
ADF–Fisher Chi-square					
Level	58.14 (0.32)	63.89 (0.16)	102.03 (0.00)	55.07 (0.43)	31.38 (0.99)
First diff	195.74 (0.00)	210.57 (0.00)		200.74 (0.00)	146.32 (0.00)
PP–Fisher Chi-square					
Level	43.73 (0.83)	59.63 (0.27)	98.54 (0.00)	39.16 (0.93)	32.42 (0.99)
First diff	260.14 (0.00)	265.57 (0.00)		276.19 (0.00)	171.68 (0.00)
The order of integration	I(1)	I(1)	I(0)	I(1)	I(1)

Note: The *p*-values are given in parentheses. In the test equation, we included the intercept and trend. Source: own computations using EViews 12.

**Table 5:** Panel cointegration test results.

	Model 1 ARRIV-ENV		Model 2 ARRIV-HEALTH		Model 3 REC-ENV		Model 4 REC-HEALTH	
	Statistic	*p*-Value	Statistic	*p*-Value	Statistic	*p*-Value	Statistic	*p*-Value
Within-dimension								
Panel v-statistic	3.683625	0.0001	3.876197	0.0001	−0.210667	0.5834	−1.145637	0.8740
Panel rho-statistic	1.419419	0.9221	1.416240	0.9216	0.182504	0.4276	1.270600	0.8981
Panel PP-statistic	−1.918058	0.0318	−1.791743	0.0420	−5.228554	0.0000	−2.540747	0.0055
Panel ADF-statistic	−4.054813	0.0000	−1.727418	0.0482	−7.280367	0.0000	−1.705050	0.0441
Between-dimension								
Group rho-statistic	2.978761	0.9986	2.999355	0.9986	0.939224	0.8262	1.326143	0.9076
Group PP-statistic	−1.907111	0.0341	−1.609483	0.0500	−9.287462	0.0000	−10.65878	0.0000
Group ADF-statistic	−3.033485	0.0012	−2.801213	0.0025	−9.161205	0.0000	−7.304295	0.0000

Source: own computations using EViews 12.

**Table 6:** ARDL panel estimation. ARRIV—endogenous variable.

Dependent Variable: ARRIV				
	Model 1—ENV		Model 2—HEALTH	
	*Coefficient*	*Prob.*	*Coefficient*	*Prob.*
Long-Term Coefficients				
lnHAPP	6.259564 ***	0.0000	9.76588 ***	0.0000
lnENV	2.505574 ***	0.0000		
lnHEALTH			7.281565 ***	0.0000
LnHAPP * lnENV	−0.992680 ***	0.0000		
LnHAPP * lnHEALTH			−3.759633 ***	0.0000
Short-Term Coefficients				
ECT	−0.028638 *	0.0744	−0.040671 **	0.0551
D(lnHAPP)	−5.260931	0.2631	−6.049136	0.1402
D(lnENV)	−1.606622	0.1837		
D(lnHEALTH)			−1.530984	0.1245
D(LnHAPP * lnENV)	0.804470	0.1919		
D(LnHAPP * lnHEALTH)			0.795979	0.1244
Intercept	−0.300319 *	0.0994	−1.493536 **	0.0592

*, ** and *** denote rejection of the null hypothesis at the 0.10 level, 0.05 level and 0.01 level, respectively. Source: own computations using EViews 12.

**Table 7:** ARDL panel estimation. REC—endogenous variable.

Dependent Variable: REC				
	Model 3—ENV		Model 4—HEALTH	
	*Coefficient*	*Prob.*	*Coefficient*	*Prob.*
Long-Term Coefficients				
lnHAPP	9.524254 ***	0.0000	3.692108 **	0.0106
lnENV	2.636824 ***	0.0000		
lnHEALTH			2.016020 ***	0.0000
LnHAPP * lnENV	−1.036123 ***	0.0000		
LnHAPP * lnHEALTH			−0.665110 ***	0.0009
Short-Term Coefficients				
ECT	−0.087741 ***	0.0022	−0.254389 ***	0.0000
D(lnHAPP)	−3.451117	0.5006	−10.22447 *	0.0475
D(lnENV)	−0.680245	0.6302		
D(lnHEALTH)			−2.070502 *	0.0999
D(LnHAPP * lnENV)	0.523999	0.4850		
D(LnHAPP * lnHEALTH)			1.397038 **	0.0348
Intercept			2.578971 ***	0.0000

*, ** and *** denote rejection of the null hypothesis at the 0.10 level, 0.05 level and 0.01 level, respectively. Source: own computations using EViews 12.

## Appendix A

**Table A1:** Cross-section short-term coefficients. ARRIV—dependent variable.

Dependent Variable: International Tourism Measured by the Number of Arrivals								
Country	Model 1				Model 2			
	ECT	HAPP	ENV	HAPP × ENV	ECT	HAPP	HEALTH	HAPP × HEALTH
		Short-term coefficients				Short-term coefficients		
Austria	0.03 ***	−0.35	−0.07	0.02	0.03 ***	2.66	0.69	−0.34
Belgium	0.03 ***	6.66	1.84	−0.89 *	0.03 ***	4.45	1.31	−0.64
Bulgaria	0.07 ***	6.09	1.45 *	−1.03 **	−0.01 ***	0.18	0.45	−0.06
Cyprus	−0.07 ***	−1.41	−0.74	0.32	0.05 ***	2.24	0.42	−0.29
Denmark	0.08 ***	5.05	1.22	−0.48	0.08 ***	22.05	5.23	−2.45
Czech Rep.	−0.01 **	10.61	2.41	−1.37	−0.01	9.55	2.17	−1.30
Croatia	−0.16 ***	−29.92	−9.26	5.19	−0.16 ***	−28.98	−7.66	4.33
Estonia	−0.02 ***	1.69	0.51	−0.29 *	−0.04 ***	2.74	0.70	−0.40 *
Finland	−0.19 ***	−7.05	−2.07	0.98	−0.09 ***	−6.44	−1.52	0.71
France	0.02 ***	24.37	4.59 *	−2.40 **	0.05 ***	24.12	5.52 *	−2.85 **
Greece	−0.15 ***	−6.99	−1.64 ***	1.04 ***	−0.07 ***	−7.36	−2.43 *	1.16 **
Germany	0.01 ***	33.72	6.60	−3.45	0.01 ***	10.36	2.36	−1.24
Hungary	0.04 ***	−0.82	−0.23	0.12	−0.22 ***	−2.75	−0.84	0.37
Ireland	−0.008 **	0.94	0.33	−0.14	0.007 ***	−7.43	−1.69	0.86
Italy	−0.01 ***	−12.04	−2.23	1.23	0.08 ***	−4.81	−1.12	0.58
Lithuania	−0.01 ***	−1.40	−0.58 **	0.34 ***	−0.004 **	−5.68	−1.42 **	0.90 **
Luxembourg	−0.02 ***	−11.20	−4.10	1.98 *	−0.08 ***	−49.91	−12.11	5.87
Latvia	−0.22 ***	−1.08	−0.21	0.16	−0.33 ***	−5.45	−1.32	0.89
Malta	0.08 ***	0.67	0.36	−0.18	0.07 ***	2.49	1.05	−0.41
Nederland	0.01 ***	−39.97	−9.22	4.36	0.01 ***	−22.28	−5.93	2.75
Poland	−0.09 ***	−21.24	−4.90	2.69	−0.34 ***	13.29	3.62	−2.07
Portugal	−0.05 ***	8.05	1.66	−1.02	−0.009	−0.61	−0.78	0.15
Romania	0.05 ***	−4.44	−1.45 ***	0.88 ***	−0.006 **	−5.54	−1.56 *	1.15 ***
Slovakia	−0.07 ***	8.92	2.45	−1.52 *	−0.03 ***	7.86	2.11	−1.30 *
Slovenia	−0.004	−4.59	−1.80	0.91	0.009 ***	−42.86	−11.24	5.94
Spain	−0.01 ***	−2.62	−0.46	0.28	−0.02 ***	−2.78	−0.59	0.35
Sweden	−0.06 ***	−103.6	−27.82	13.97	−0.006 *	−72.42	−16.74	8.81

Note: ***, ** and * denote that the coefficient is significant at the level of 0.01 (2-tailed), 0.05 (2-tailed) and 0.10 (2-tailed).

**Table A2:** Cross-section short-term coefficients. REC—dependent variable.

Dependent Variable: International Tourism Measured by Receipts in Current USD								
Country	Model 3				Model 4			
	ECT	HAPP	ENV	HAPP × ENV	ECT	HAPP	HEALTH	HAPP × HEALTH
		Short-term coefficients				Short-term coefficients		
Austria	0.04 ***	−11.83	−2.56	1.53	0.04 **	2.20	1.41	−0.28
Belgium	0.02 ***	0.97	0.32	0.15	−0.11 **	−5.85	−1.07	0.92
Bulgaria	0.04 ***	4.82	1.25	−0.78	−0.51 ***	15.44	4.80	−2.52
Cyprus	0.02 ***	0.93	0.37	−0.19	−0.27 ***	−6.48	−1.06	0.90
Denmark	−0.05 ***	−60.86	−17.05	8.18	−0.02	−17.78	−3.58	1.93
Czech Rep.	−0.08 **	11.37	2.95	−1.46	−0.26 ***	10.12	3.11	−1.38
Croatia	0.01 ***	−80.02	−22.89	13.43	−0.39 ***	−37.43	−9.19	5.52
Estonia	0.03 ***	−3.03	−1.83	1.10	−0.26 ***	−1.99	−0.17	0.53
Finland	−0.07 ***	45.14	14.75	−7.18	−0.04	−35.80	−7.00	4.16
France	−0.38 ***	27.37	5.40	−2.72	−0.30 ***	15.83	3.89	−1.89
Greece	0.03 ***	−0.08	0.02	0.14	−0.20 ***	−5.60	−1.54	0.96 *
Germany	−0.17 ***	25.73	5.50	−2.59	−0.03 **	21.36	5.70	−2.56
Hungary	0.04 ***	11.03	2.81	−1.61	−0.13 ***	−0.91	−0.10	0.20
Ireland	−0.08 **	13.89	4.11	−1.91	−0.004	−1.05	0.29	0.13
Italy	−0.05 ***	−4.95	−0.41	0.51	−0.11 ***	−3.91	−0.20	0.51
Lithuania	0.01 ***	3.17	0.78	−0.42	−0.21 ***	9.61	2.99*	−1.36 **
Luxemburg	−0.32 ***	−5.39	−2.13	0.97	−0.27 ***	−99.32	−23.19	11.59
Latvia	−0.07 ***	−3.22	−1.30	0.83	−0.32 ***	−2.48	−0.76	0.73
Malta	−0.05 ***	3.89	1.47	−0.74	−0.54 ***	3.15	1.13	−0.36
Nederland	−0.06 ***	−52.73	−10.68	5.25	−0.13 ***	−5.48	0.09	0.19
Poland	−0.36 ***	−8.61	−1.89	1.13	−0.73 ***	−48.88	−12.82	7.72
Portugal	0.05 ***	−11.88	−2.18	1.70	−0.01 ***	2.70	1.14	−0.32
Romania	0.01 *	−5.36	−1.37	1.16	−0.33 ***	−7.01	−1.72	1.44 *
Slovakia	−0.19 ***	−4.66	−1.16	0.64	−0.45 ***	−12.17	−2.88	1.82
Slovenia	−0.12 ***	32.83	11.39	−5.88	−0.01 **	−6.88	−0.95	1.00
Spain	−0.49 ***	−17.70	−2.61	2.11	−0.92 ***	−66.90	−16.06	8.98
Sweden	−0.10 ***	−4.01	−1.44	0.78	−0.23 ***	9.49	1.86	−0.86

Note: ***, ** and * denote that the coefficient is significant at the level of 0.01 (2-tailed), 0.05 (2-tailed) and 0.10 (2-tailed).
